# Temporal Variation of b Value with Statistical Test in Wenchuan Area, China Prior to the 2008 Wenchuan Earthquake

**DOI:** 10.3390/e24040494

**Published:** 2022-03-31

**Authors:** Weiyun Xie, Katsumi Hattori, Peng Han, Haixia Shi

**Affiliations:** 1 Graduate School of Science and Engineering, Chiba University, Chiba 263-8522, Japan; xieweiyun945@gmail.com; 2 Graduate School of Science, Chiba University, Chiba 263-8522, Japan; 3 Center for Environmental Remote Sensing, Chiba University, Chiba 263-8522, Japan; 4 Research Institute of Disaster Medicine, Chiba University, Chiba 263-8522, Japan; 5 Department of Earth and Space Sciences, Southern University of Science and Technology, Shenzhen 518055, China; hanp@sustech.edu.cn; 6 China Earthquake Networks Center, Beijing 100045, China; shihaixia08@seis.ac.cn

**Keywords:** b value, Mc, AIC, bootstrap, Gutenberg–Richter

## Abstract

The Gutenberg–Richter b value describes the ratio between large and small events. A number of studies have suggested that the b value decreases before large earthquakes. In this study, we investigate the temporal variation of the b value of an area along the main rupture zone of the 2008 Wenchuan earthquake (M8.0) prior to the great event. Before estimating b values, we tested the earthquake catalog to make sure that we use the reliable frequency–magnitude distribution by the calculation of MC (completeness of magnitude). We define parameter P (ΔAIC ≧ 2) values to examine the significance level of b-value changes in the temporal variation by combining a boostrap method with Akaike’s Information Criterion (AIC). The b value in the main rupture zone shows a long-term decrease trend. We then focus on a smaller area where the initial rupture starts. The results show that b values significantly changed about 3 months before the 2008 Wenchuan earthquake in the initial rupture area, indicating that the b value has a potential capability to monitor and detect precursory phenomena of great earthquakes.

## 1. Introduction

Gutenberg and Richter [1] put forward the Gutenberg–Richter law (hereafter, GR law),

log_10_N = a − bM, (1)

which denotes the relationship between magnitude (M) and number (N) of earthquakes. Here, the constant a value measures the productivity of earthquake, and the b value is the relative proportion which represents the seismicity (e.g., [2]). Later studies showed that the temporal and spatial changes in the b value were capable of reflecting the stress evolution around seismogenic zones (e.g., [3,4,5]). A number of reports demonstrated decreases in the b value prior to great earthquakes in recent decades, such as the 2003 Tokachi-Oki earthquake (Mw8.3) [6]; the 2004 Sumatra earthquake (Mw9.1) [7]; the 2011 earthquake off the Pacific coast of Tohoku (Mw9.0) [8]; the 2014 Kumamoto earthquake (M7.3) [9]; the 2019 Ridgecrest earthquake [10]; and the assessment of the earthquake forecast in Yunnan, China [11]. This decrease was discussed in Varotsos et al. [12] on the basis of natural time analysis, according to which the temporal correlations between earthquake magnitudes, as well the variability of the order parameter of seismicity, are affected [13,14] before major earthquakes whose epicenters can be estimated well in advance [15]. Changes in b value were used to discriminate between foreshocks and aftershocks, and a traffic-light classification was proposed for the real-time assessment of the probability of a subsequent larger event [16]. The experimental study in (Scholz [17]; Lei [18]) also suggested that variation of the b value in rock samples has a decrease trend before the main rupture. The completeness of magnitude (Mc) of the earthquake catalog is the minimum magnitude of earthquakes, above which the earthquake distribution follows the G-R law, and the all eartquakes are considered to be recorded completely. Mc is essential to calculate the b value. Several methods of its estimation are compared in Woessner and Wiemer [19]. After computation of the b value, the variation should be evaluated objectively to figure out whether there is precursory decrease in the b value prior to a great earthquake. Some geophysical data such as geomagnetic data, satellite thermal infrared data, strain data, and GNSS ionospheric electron data show the precursor characteristics based on the investigations on statistical significance using superposed epoch analysis and assessment of pre-earthquake phenomena using ROC approaches [20,21,22,23,24,25,26,27,28]. For the reason of uncertainty in the b value, we employed the Utsu test [29] which is shown in Schorlemmer et al. [30], to quantify the significance level of b-value changes.

In this study, we investigate the temporal variation of b value of an area along the main rupture zone of the 2008 Wenchuan earthquake (M8.0) prior to the great event. We define the “reference seismicity” as the seismicity at the beginning of the data (2000–2003) and compare the seismicity afterwards with it by statistical test to see how the seismicity changed with time before the great event.

## 2. Data

**Figure 1:**
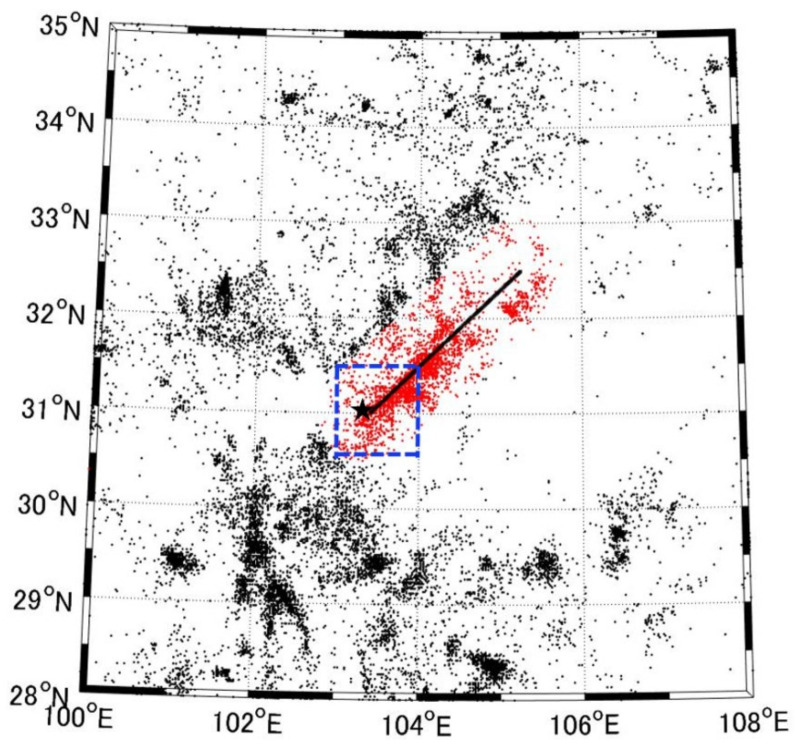
Spatial distribution of earthquake events during 1 January 2000–12 May 2008. The black star is the epicenter of the 2008 Wenchuan earthquake, and the black solid line shows the main rupture zone. The red spots show the earthquakes that occurred in area 1, and the blue dotted frame indicates area 2.

In order to analyze the 2008 Wenchuan earthquake, we used a dataset from the China Earthquake Administration (CEA) earthquake catalog dating from 2000 to 2013. For the dataset, we used a depth range of 0–60 km and two different areas: a broader area and a smaller area. The broader region is defined by the main rupture zone and is within 60 km of the main fault, which is approximated to the line from (31.00° N, 103.40° E) to (32.5° N, 105.25° E) with reference to He and Shen [31]. We also investigated another smaller area, which corresponds to a rectangular region between (30.5° N–31.5° N) and (103° E–104° E), where the initial rupture of the 2008 Wenchuan earthquake started. Figure 1 shows the spatial distribution of earthquake events during 1 January 2000–12 May 2008 in areas 1 and 2.

## 3. Methods

### 3.1. Estimation of MC

Because of detection capability, weak earthquakes are not recorded completely, which leads to a deviation in frequency of magnitude distribution. To calculate b values correctly, we have to use the distribution without missing earthquakes. We chose the maximum MC for the whole analyzed period. Regarding the MC computations, we divided the earthquake catalog into windows, with each window having the same number of earthquakes. The window then shifted in step chronologically. The step can be constant in number of earthquakes or time. In this study, we set the window number to 500, step to 50 earthquakes, and used MAXC (maximum curvature) technique Wiener and Wyss [32] to estimate the MC for each window. We then applied the bootstrap approach on each window (with replacement) and repeated 300 times to obtain 300 Mcs. We took the mean of Mc as the Mc estimation and the standard deviation as the Mc error for each window.

### 3.2. Estimation of b Values

We employed the maximum-likelihood method to calculate b values [33,34]. According to Aki [33], the b value and its error $\sigma_{b}$ can be calculated in Equations (2) and (3):

(2) $$b=\frac{1}{\log10\left({\bar{M}}_{i}-\mathrm{Mc}\right)}$$

(3) $$\sigma_{b}=\frac{b}{\sqrt{N}}$$

where N is the earthquake number, and ${\bar{M}}_{i}$ is the mean value of earthquake magnitudes. We used two different types of step to calculate b values. The first type took a certain number of earthquakes (50 or 100) as a step to investigate the temporal variation of b values. In the second type, we took a certain time interval to define a step, which allowed us to investigate the daily variation and the monthly variation of b values.

Nava et al. [35] showed estimates on the probabilities of obtaining correct estimates of b for a given desired precision for samples of different sizes. It was suggested that when N = 500 or more, the standard deviation of b value could be less than 5% (when true b = 1.0). Therefore, in this study, N was set at 500. When shifting by time interval, the window was obtained by setting the time at the end of the day/month and searching back until there were 500 earthquakes.

### 3.3. Significance Level of b-Value Changes

We applied the AIC (Akaike’s Information Criterion) to define the significance level of difference between two b values. ΔAIC ≧ 2 indicates a significant difference between b values in two different time windows, referring to the previous research [29]:

(4) $$∆\mathrm{AIC}=-2\left(2N\right)\ln\left(2N\right)+2Nln\left(N+N\frac{b_{1}}{b_{2}}\right)+2Nln\left(N+N\frac{b_{2}}{b_{1}}\right)-2$$

where *N* is the number of samples, and *b*₁ and *b*₂ are b values in two different windows for testing. In order to obtain a robust reference b value, we applied the bootstrap approach (with replacement) in the reference seismicity period (2000–2003) and sample of 5000 windows (N = 500 in each window) and computed b values of each window to generate the reference b-value group. We then calculated ΔAIC to quantify the differences between the b value to test and each b value in the reference group, which contained 5000 b values. We counted the number of ΔAIC ≧ 2 in 5000 ΔAIC values and defined the percentage as P (ΔAIC ≧ 2). By these means, we evaluated the significance of b-value temporal changes. A larger P indicates a more significant difference between the b value and the reference.

## 4. Results

**Figure 2:**
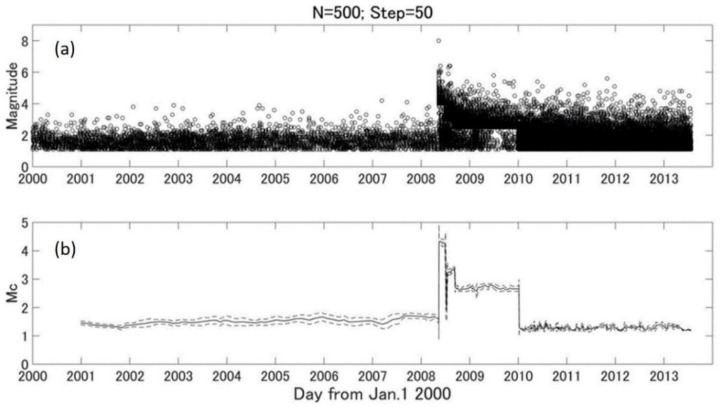
The temporal distribution of earthquakes and variations of MC. (**a**) Temporal distribution of earthquake events; (**b**) a solid line shows the temporal variation of MC, and the broken lines indicate the standard deviation of MC for N = 500 and step = 50.

According to the report of Liu et al. [36], the China Digital Seismological Observational System, including the national and regional seismic stations, was built from 1996–2000 by the China Earthquake Administration (CEA). The establishment of regional telemetered digital-seismograph networks by the end of 2000 significantly improved the capability of monitoring regional seismicity. Figure 2 shows the result of MC estimation. It indicates a quick increase after the 2008 Wenchuan earthquake (M8.0). As shown in Equation (2), the b value is sensitive to the variation of Mc value. To keep a relatively stable Mc, we chose the dataset from 1 January 2000 until the main shock of the 2008 Wenchuan earthquake. Taking the maximum Mc (1.7) during this period, there were 1930 earthquakes for area 1 and 909 earthquakes for area 2, respectively.

**Figure 3:**
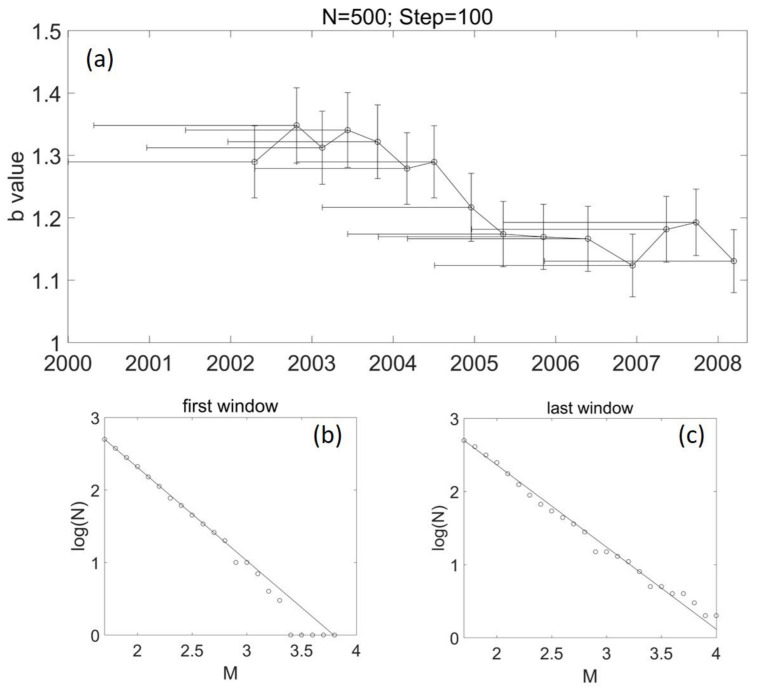
The temporal variation of b values in area 1 for N = 500 and step = 100. (**a**) The vertical error bar shows the standard deviation of the b value; the horizontal error bar presents the corresponding time span of the window. (**b**) Frequency–magnitude distribution of earthquakes in the first window in (**a**). The window starts from 2 January 2000 (first EQ in area 1) and terminates on 19 March 2002; the b value is 1.29 ± 0.058. (**c**) Frequency–magnitude distribution of earthquakes in the last window in (**a**). The window starts from 8 November 2005 and terminates on 13 March 2008; the b value is 1.13 ± 0.05.

Figure 3a shows the temporal variation of b values in the main rupture zone (area 1) of the 2008 Wenchuan earthquake. It was estimated with a window size of 500 events and a shifting step of 100 samples. The temporal variation shows that the b value changed a little from 2002 to the middle of 2004. After that, the b value presented a tendency to decrease.

**Figure 4:**
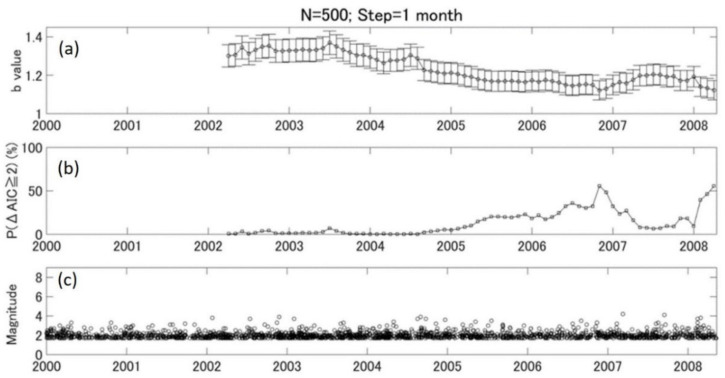
The monthly variation of b values, *p* values, and earthquake distributions in area 1. (**a**) The monthly variation of b values with N = 500. The step of shifting window is 1 month. The vertical error bar shows the standard deviation of the b value. (**b**) The temporal variation of P (ΔAIC ≧ 2) value. (**c**) The temporal distribution of earthquakes with M ≥ Mc.

We then evaluated the monthly variation of b value relative to background (2000–2003) using a P (ΔAIC ≧ 2) parameter. The results in Figure 4 were obtained with window size N = 500 and step = 1 month. The monthly b value presents a similar tendency of variation, as shown in Figure 3. The significance level of b-value changes shows two peaks around the end of 2006 and few months before the Wenchuan earthquake in 2008. We looked further into the second peak and found that most events from February to April 2008 were concentrated at the southwest side of the main rupture zone, where the initial rupture of the M8.0 earthquake started. Therefore, we narrowed the analysis area to area 2 to see if there was any difference in b-value temporal variation.

**Figure 5:**
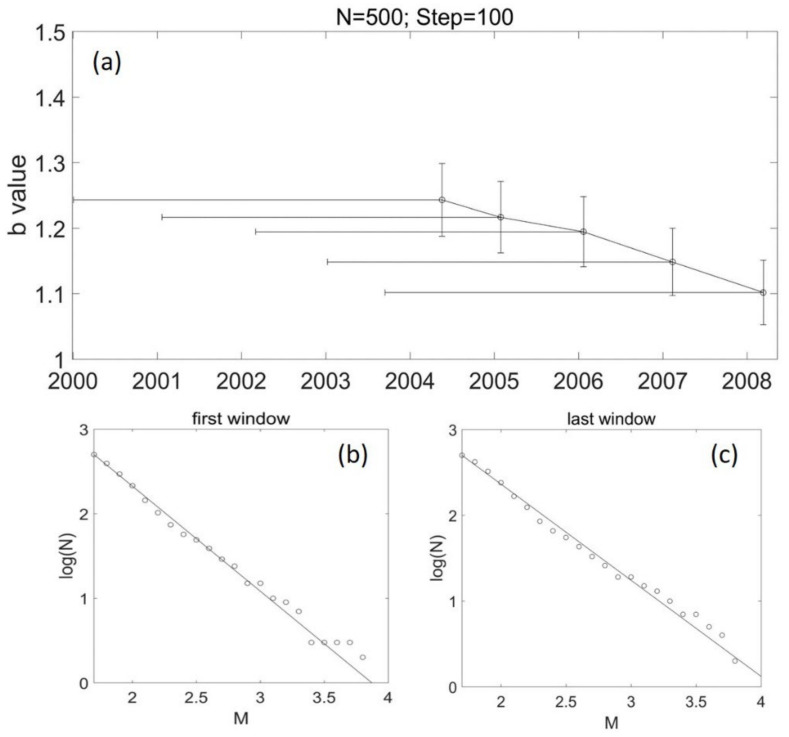
The temporal variation of b values in area 2 for N = 500 and step = 100. The vertical error bar shows the standard deviation of the b value; the horizontal error bar presents the corresponding time span of the window. (**b**) Frequency–magnitude distribution of earthquakes in the first window in (**a**). The window starts from 4 January 2000 (the first EQ in area 2) and terminates on 19 May 2004; the b value is 1.24 ± 0.06. (**c**) Frequency–magnitude distribution of earthquakes in the last window in (**a**). The window starts from 14 September 2003 and terminates on 12 March 2008; the b value is 1.10 ± 0.05.

**Figure 6:**
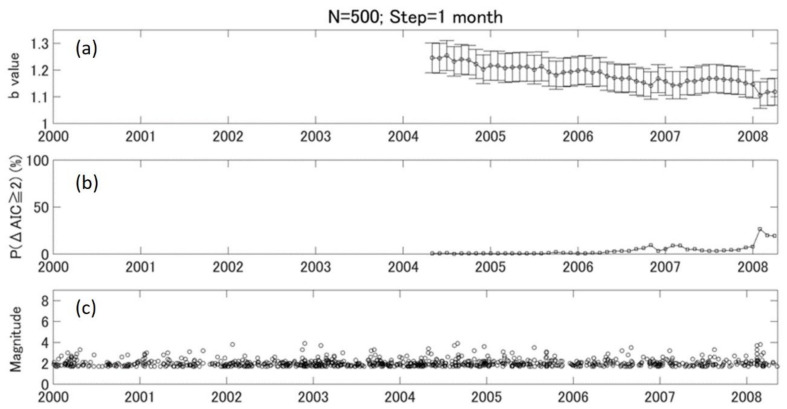
The monthly variation of b values, *p* values, and earthquake distributions in area 2. (**a**) The monthly variation of b values with N = 500. The step of shifting window is 1 month. The vertical error bar shows the standard deviation of the b value. (**b**) The temporal variation of P (ΔAIC ≧ 2) value. (**c**) The temporal distribution of earthquakes with M ≥ Mc.

**Figure 7:**
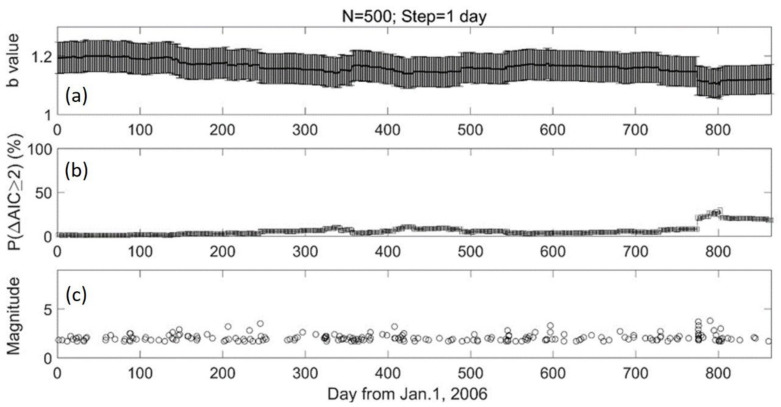
The daily variation of b values in area 2. The lateral shows the day, from 1 January 2006 till 11 May 2008. (**a**) The daily variation of b values with N = 500. The step of shifting window is 1 day. The vertical error bar shows the standard deviation of the b value. (**b**) The temporal variation of P (ΔAIC ≧ 2) value. (**c**) The temporal distribution of earthquakes with M ≥ Mc.

Figure 5 presents the temporal variation of b values in area 2 for N = 500 and step = 100. Though there were only 5 windows due to the low number of earthquake events, we could still find a decrease trend in the b value. Figure 6 shows the monthly variation of b values, *p* values, and earthquake distributions in area 2. There was only one dramatic increase about 3 months before the 2008 Wenchuan earthquake. To evaluate whether the b value could serve as a short-term precursor, we then computed the daily b-value variation. Figure 7 shows the detailed results of the daily variations in area 2 from 1 January 2006 to 11 May 2008, with N = 500 and shifting window = 1 day. It was found that at the beginning of February 2008, the *p* value increased suddenly and stayed at a relatively high value of 20%.

## 5. Discussion

### 5.1. Mc Estimation

The MAXC method may underestimate the Mc. Woessner and Wiemer [19] compared four different methods for Mc estimation (EMR, MAXC, GFT, and MBS); the results suggested that EMR had a superior performance. However, when the sample size was large—for example, more than several hundred—the estimations of Mc using different approaches were quite similar. The errors were mostly around 0.1. In our computation, to make sure the Mc was not underestimated, we took Mc + 0.1 as the Mc output in our program. We also computed the b value using Mc estimated with the GFT method; the results were similar as those using the MAXC method. Moreover, it has to be emphasized that the b value could change when using different Mc, but the b-value variation trend is usually not affected by Mc.

### 5.2. b-Value Calculation and the Significance of b-Value Change

According to Utsu [34], if the binning is 0.1 magnitude unit, the Mo should be Mc-0.05, which will lead to a slight decrease in b value compared with Equation (2) proposed by Aki [33]. In the real case, it is very difficult to estimate the binned magnitude. Moreover, what we concern here is the b value’s evolution rather than the absolute b value. The b values obtained by Utsu [34] and Aki [33] are slightly different; however, the b-value temporal changes are almost the same. Shi and Bolt [37] proposed an equation for b-value error estimation. Compared with the equation proposed by Aki [33], it considered the differences between each magnitude with mean magnitude to estimate the error of b value. When the number of earthquakes N was large—for example, N = 500 in this study—the errors estimated by equations of Aki [33] and Shi and Bolt [37] were almost the same.

Stumpf and Porter [38] recently pointed out that one needs two orders of magnitude in both axes to truly establish a power law. We investigated the frequency–magnitude distributions in each window in Figure 3 and Figure 5. It was found that the b value fits the data well, and there are two orders of magnitude in both axes, suggesting the b-value estimation is robust.

As the b value is a statistical parameter and it has an uncertainty, the key point is that one has to make sure that the change in b value is significant. To achieve this, we took 2000–2003 as a reference period and applied the bootstrap approach (with replacement) to generate the reference b-value group. The ΔAIC test proposed by Utsu [29] was then employed to quantify the significance level of b-value changes. In fact, the cycle of an M8 class earthquake could be more than hundred years, and our data length is far from enough to obtain a “normal seismicity” background. In this study, what we could see was just the stress evolution prior to the M8 event by inferring from the b-value variation. We took the beginning of the data as a reference period and compared the seismicity afterwards to see how the seismicity changed with time before the great event. By these means, we might obtain some useful information on the stress evolution before great earthquakes.

### 5.3. b Value and Stress Evolution

Scholz [3] showed that the b value for earthquakes decreases linearly with stress for both continental and subduction-zone environments. He found that b = 1.23 ± 0.06–(0.0012 ± 0.0003) (σ1–σ3) using linear-regression analysis. In fact, other factors such as material heterogeneity and thermal gradients (swarms) could also affect b values. We agree that the b value for earthquakes decreases with stress, but we cannot agree with the quantitative relation without considering the effects of other variables. If we consider the relation between b value and stress in a given area, then the results might be more robust, as the material heterogeneity and other factors could be regarded as constant. Tan et al. [5] showed that the b value decreases with increasing tidal stress in a 25 km^3^ block of crust that experiences periodic tidal loading of ±20 kPa. They found that b = 1.35 ± 0.03 − (0.007 ± 0.003) σ where stress is in kPa. In our results, the b-value change from 2002–2008 was about 0.1–0.2 (See Figure 3, Figure 4, Figure 5 and Figure 6). If using the relation of Tan et al. [5], the stress change is about tens of kPa. In our opinion, the relation between stress and b value may not be linear. When the stress level is subcritical, there might be one relation; when the stress level becomes critical, a small increase in stress may lead to a dramatic decrease in b value. Moreover, as mentioned before, material heterogeneity and the fractal dimension of the fault system may affect the relation. Therefore, different places may show different relations between stress changes and b values. However, the decrease in b value, as seen in this study and many other cases, might be a common phenomenon prior to large earthquakes, suggesting there is stress accumulating in the earthquake-preparation process.

### 5.4. b Value for Earthquake Forecast

The temporal variation of b values in both the whole rupture zone (area 1) and the initial rupture area (area 2) shows a clear decrease trend, implying that the b value contains long-term precursory information. As for the monthly and daily variations, there are clear peaks in *p* value a few weeks before the main shock, suggesting that the b-value change is significant and has a potential capability of a middle-to-long term forecast of an impending great earthquake.

In order to achieve complete recognition of b-value changes in the preparation process of the 2008 Wenchuan earthquake, further investigation is required to analyze the spatial distribution of b values in the Wenchuan area in the future. For the purpose of earthquake forecast, b values should be integrated with other parameters such as radon concentration, GPS deformation, groundwater level, and so on, to achieve more acute results.

## 6. Conclusions

In this study, we investigated the temporal variation of the b value prior to the 2008 Wenchuan earthquake (M8.0). We proposed a new parameter P (ΔAIC ≧ 2) to examine the significance level of b-value changes in the temporal variation by combining a bootstrap method with Akaike’s Information Criterion (AIC). The b values in both the main rupture zone and initial rupture area showed a long-term decrease trend. Monthly and daily variations showed that b values significantly decrease about 3 months before the main shock in the initial rupture area, indicating that the b value has the potential capability to monitor large earthquakes.

## Data Availability

The earthquake catalogs are provided by the China Earthquake Networks Center.
